# *RASSF1A* hypermethylation in pretreatment serum DNA of neuroblastoma patients: a prognostic marker

**DOI:** 10.1038/sj.bjc.6604887

**Published:** 2009-01-22

**Authors:** A Misawa, S Tanaka, S Yagyu, K Tsuchiya, T Iehara, T Sugimoto, H Hosoi

**Affiliations:** 1 Department of Pediatrics, Kyoto Prefectural University of Medicine, Kyoto, Japan; 2 Department of Clinical Trial Design and Management, Translational Research Center, Kyoto University Hospital, Kyoto, Japan

**Keywords:** *RASSF1A*, methylation, serum, DNA, neuroblastoma

## Abstract

The tumour suppressor gene *RASSF1A* is known to be frequently silenced by promoter hypermethylation in neuroblastoma tumours. Here we explored the possible prognostic significance of aberrant promoter hypermethylation of *RASSF1A* in serum DNA samples of patients with neuroblastoma as a surrogate marker for circulating tumour cells. We analysed the methylation status of the *RASSF1A* gene in matched tumour and pretreatment serum DNA obtained from 68 neuroblastoma patients. Hypermethylation of *RASSF1A* in tumour samples was found in 64 patients (94%). In contrast, serum methylation of *RASSF1A* was observed in 17 patients (25%). Serum methylation of *RASSF1A* was found to be statistically associated with age ⩾12 months at diagnosis (*P*=0.002), stage 4 (*P*<0.001) and *MYCN* amplification (*P*<0.001). The influence of serum *RASSF1A* methylation on prognosis was found to be comparable with that of the currently most reliable marker, *MYCN* amplification on univariate analysis (hazard ratio, 9.2; 95% confidence interval (CI), 2.8–30.1; *P*<0.001). In multivariate analysis of survival, methylation of *RASSF1A* in serum had a hazard ratio of 2.4 (95% CI, 0.6–9.2), although this association did not reach statistical significance (*P*=0.194). These findings show that the methylation status of *RASSF1A* in the serum of patients with neuroblastoma has the potential to become a prognostic predictor of outcome.

Neuroblastoma is the most common extracranial solid tumour in children and is characterised by a wide range of clinical behaviours, from spontaneous regression to rapid progression with a fatal outcome (Maris *et al*, 2007). The clinical outcome is associated with disease stage, age at diagnosis, *MYCN* amplification and histological classification. Although numerous genetic abnormalities, including *MYCN* amplification, are associated with tumour progression and poor outcome, the molecular mechanisms responsible for the pathogenesis of aggressive neuroblastoma remain unclear. Identifying such molecular changes may contribute to improved clinical management and outcome prediction of newly diagnosed neuroblastomas.

In recent years, changes in the status of DNA methylation, known as epigenetic alterations, have turned out to be one of the most common molecular alterations in human neoplasia including neuroblastoma (Misawa *et al*, 2005; Sugino *et al*, 2007). Several potential tumour-suppressor genes have been described as frequently silenced by hypermethylation in neuroblastomas. Methylation of promoter CpG islands is known to inhibit transcriptional initiation and cause permanent silencing of downstream genes. Loss of heterozygosity of chromosome 3p21.3 is one of the most frequent alterations in solid tumours. Located within this 3p21.3 locus, the RAS-association domain family 1, isoform A gene (*RASSF1A*) encodes a RAS effector that has been identified as a tumour suppressor of many different cancer types (Dammann *et al*, 2000). *RASSF1A* falls into the category of genes frequently inactivated by methylation rather than mutational events. This gene is silenced and inactivated by promoter region hypermethylation in many adult and childhood cancers, including neuroblastoma (Astuti *et al*, 2001; Harada *et al*, 2002; Wong *et al*, 2004; Yang *et al*, 2004; Banelli *et al*, 2005; Lazcoz *et al*, 2006; Michalowski *et al*, 2008). *RASSF1A* has been shown to play important roles in cell cycle regulation, apoptosis and microtubule stability as a tumour suppressor gene (Agathanggelou *et al*, 2005).

It is well known that DNA fragments are frequently and abundantly found in the serum of cancer patients, with significantly higher levels in patients with metastasis (Hesson *et al*, 2007). A number of studies have evaluated the potential of circulating tumour-related methylated DNA in serum for the molecular diagnosis and prognosis of various types of cancer (Müller *et al*, 2003; Ibanez de Caceres *et al*, 2004; Mori *et al*, 2005). Methylation-specific PCR assay is a sensitive and specific assay for tumour-related DNA methylation in serum. Several studies have investigated the prospect of using DNA methylation as a surrogate marker for circulating tumour cells in serum samples from breast cancer or melanoma patients (Fiegl *et al*, 2005; Koyanagi *et al*, 2006). However, no studies of neuroblastoma have assayed serum samples for aberrant DNA methylation. Therefore, this study investigated whether it is possible to detect *RASSF1A* epigenetic alterations in the serum of neuroblastoma patients, and aberrant *RASSF1A* methylation in patient pretherapeutic serum is of prognostic significance in neuroblastoma using a series of matched neuroblastoma tumour and serum DNA.

## Materials and methods

### Patients and sample collection

Clinical data were collected retrospectively by reviewing the medical database at the Hospital of Kyoto Prefectural University of Medicine for the period between 1985 and 2004. After approval by the Institutional Review Board, 68 neuroblastoma patients were identified on the basis of histological examination of tumour specimens that met the following criteria: the patient had an available tumour specimen; a serum specimen was available; and the patient either died or had >1 year of follow-up time. The clinical data included information regarding tumour stage, age at diagnosis, sex, *MYCN* gene status and outcome. Staging was evaluated according to the criteria of the International Neuroblastoma Staging System (Maris *et al*, 2007). Patients of any age who had stage 1 or 2 disease and those younger than 12 months with stage 3 or 4S disease were given either surgery or surgery with chemotherapy (Matsumura and Michon, 2000). Patients aged 12 months or older with stage 3 and any patients with stage 4 disease were treated according to the protocol by the Japanese Neuroblastoma Study Group (Sawaguchi *et al*, 1990; Tsuchida and Kaneko, 2000; Kaneko *et al*, 2002; Suita *et al*, 2007). The patients with stage 4 disease underwent high-dose chemotherapy with autologous stem-cell rescue after the initial chemotherapy. Instead of pre-specified sample size determination, power analysis was conducted after collecting clinical data to guarantee statistical power and to evaluate whether *RASSF1A* methylation is a prognostic marker for survival. In a realistic scenario, a study of 68 patients had power of 96% to detect a single marker with hazard ratio larger than 5.

Tumour samples at the time of diagnosis and before the administration of chemotherapy were frozen immediately and stored at −80°C until DNA extraction. In addition, match-paired serum samples were assessed. Peripheral blood was obtained before any therapy or surgery. To avoid contamination of serum DNA by the DNA from WBCs, serum was prepared exclusively from the liquid fraction of clotted blood after centrifugation at 1000 × **g** for 10 min and stored it at −20°C until DNA extraction. For the extraction of free DNA, we used 200 *μ*l of stored serum, which supplemented with 1 *μ*g salmon testes DNA (Sigma, St Louis, MO, USA) as a carrier DNA. DNA was extracted from tumour samples using a QIAmp DNA Mini Kit and from serum samples using a QIAmp DNA Blood Mini Kit (Qiagen, Hilden, Germany) according to the manufacturer’s instructions.

### Analysis of DNA methylation

**Figure 1:**
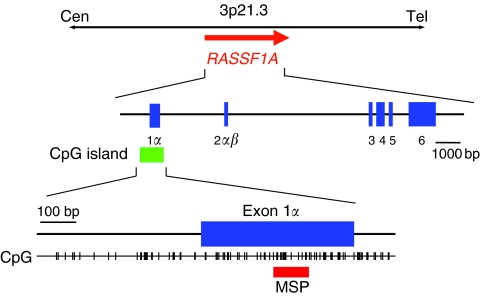
Genomic structure of the *RASSF1A* gene. Vertical tick marks, CpG sites; blue boxes, exons; green box, CpG island in the promoter; red box, region analysed by methylation-specific PCR.

Treatment of tumour DNA and serum DNA with sodium bisulphite was performed with an EZ DNA methylation kit (Zymo Research, Orange, CA, USA) following the protocol of the manufacturer. Methylation-specific PCR was performed with primers specific for either methylated or unmethylated DNA spanning the region within the *RASSF1A* gene (Figure 1). The primers used were methylation-specific RAM-1 (5′-GTGTTAACGCGTTGCGTATC-3′) and RAM-2 (5′-AACCCCGCGAACTAAAAACGA-3′) and unmethylation-specific RAU-1 (5′-TTTGGTTGGAGTGTGTTAATG-3′) and RAU-2 (5′-CAAACCCCACAAACTAAAAACAA-3′), as described earlier (Lo *et al*, 2001). PCR conditions consisted of an initial incubation for 10 min at 95°C followed by 35 cycles for tumour samples or 40 cycles for serum samples of denaturation at 95°C for 45 s, annealing at 60°C for 45 s and extension at 72°C for 60 s, followed by a final extension step of 72°C for 10 min. Lymphocyte DNA and *in vitro* methylated (using *Sss*I CpG methylase; New England Biolabs, Beverly, MA, USA) lymphocyte DNA were used as unmethylated and methylated controls, respectively. The PCR products obtained were analysed by electrophoresis in 2% agarose gels and stained with ethidium bromide. Samples were scored as methylation positive when methylated alleles were visualised as bands in the methylated DNA lane and as methylation negative when bands were seen only in the unmethylated DNA lane. The analysis of the samples in this study was performed by an analyst blinded to the clinical and biological information.

### Statistical analysis

The primary end point was overall survival defined by the period from diagnosis of the primary tumour to any cause of death. The relationship between clinicopathological variables and methylation status of the *RASSF1A* gene was shown initially using contingency tables and *χ*^2^ test. Survival curves for *RASSF1A* methylation were derived by the Kaplan–Meier method. Univariate analysis was conducted using Cox’s proportional hazard models and log-rank test. Performance of *RASSF1A* methylation as a prognostic marker was also analysed after adjustment for known prognostic factors by (i) subset analysis of stage 3 patients using contingency tables and Fisher’s exact test and (ii) multivariate Cox’s proportional hazard models including age, sex and tumour stage. Two-sided *P*-values<0.05 were considered as significant. SAS 9.13 (SAS Institute Inc., Cary, NC, USA) was used for statistical analyses.

## Results

**Figure 2:**
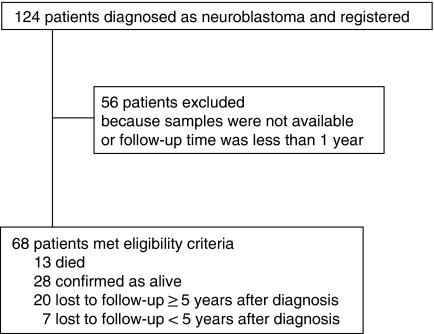
Patient disposition.

A total of 124 patients with histologically confirmed neuroblastoma or ganglioneuroblastoma were treated at the Hospital of Kyoto Prefectural University of Medicine between January 1985 and May 2004. Sixty-eight patients met the criteria of this retrospective study. The detailed patient disposition is shown in Figure 2 and the baseline characteristics of patients are presented in Table 1. Of the 68 patients, 24 were classified as stage 1, 11 as stage 2, 11 as stage 3, 18 as stage 4 and 4 as stage 4S. At the time of diagnosis, 42 patients (62%) were younger than 12 months, and 26 (38%) were older. We found no significant differences between included and excluded patients for age or stage statistically. Twelve patients (18%) had tumours with *MYCN* amplification, and *MYCN* amplification was not detected in the tumours from 56 (82%) patients by southern blot analysis or fluorescence *in situ* hybridisation. The median follow-up time was 72 months, with a range from 9 to 248 months.

### Detection of *RASSF1A* promoter methylation in tumours

This study initially investigated the hypermethylation status of the *RASSF1A* tumour suppressor genes in 68 neuroblastoma tumours. Only four (one each at stage 1, 2, 4S and 3) tumours showed no methylation of *RASSF1A* (Supplementary Table). All other neuroblastoma tumours (64 of 68; 94%) showed methylated *RASSF1A*. Hypermethylation in tumours was observed very frequently in all of the stages of neuroblastoma examined, including stage 1, 2 and 4S tumours (Supplementary Table) and no correlation between *RASSF1A* methylation and known prognostic factors including stage, age and *MYCN* amplification was detected. No relationship between *RASSF1A* methylation in tumours and outcome was also observed. *RASSF1A* methylation was not observed in any of the three benign ganglioneuromas.

### Detection of *RASSF1A* promoter methylation in serum

**Figure 3:**
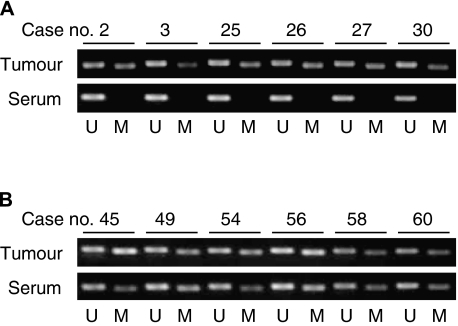
*RASSF1A* methylation status of tumour and serum DNA in neuroblastoma patients. M, methylated; U, unmethylated. The sizes of the PCR products for methylated and unmethylated primers are 93 and 105 bp, respectively. (**A**) Cases stage 1 and 2 with good prognosis tumour DNAs are methylated but absent in the serum DNAs. (**B**) In contrast, in stage 3 and 4 *MYCN*-amplified cases, methylated DNAs are detected in both tumour and serum samples.

The hypermethylation status of *RASSF1A* in the matched serum DNA samples was then determined and compared with the pattern of hypermethylation found in the corresponding tumour DNA samples (Figure 3). *RASSF1A* hypermethylation was detected in 17 of 68 (25%) matched serum DNA samples (Table 1). The detailed overview is shown in Supplementary Table.

### Correlation of serum *RASSF1A* methylation status with clinical factors

The methylation status of *RASSF1A* in the pretherapeutic serum of the 68 patients was analysed for association with known prognostic factors (Table 2). Serum *RASSF1A* methylation showed a significant statistical association with age ⩾12 months (*P*=0.002). *RASSF1A* methylation in serum was detected more frequently in disseminated stage 4 tumours than local-regional (stage 1, 2 and 3) and 4S tumours (*P*<0.001). Furthermore, serum-methylated *RASSF1A* was significantly correlated with *MYCN* amplification (*P*<0.001). Notably, all cases with *MYCN* amplification showed *RASSF1A* methylation of serum DNA (Supplementary Table).

### Analysis of prognostic significance of *RASSF1A* methylation in serum

**Figure 4:**
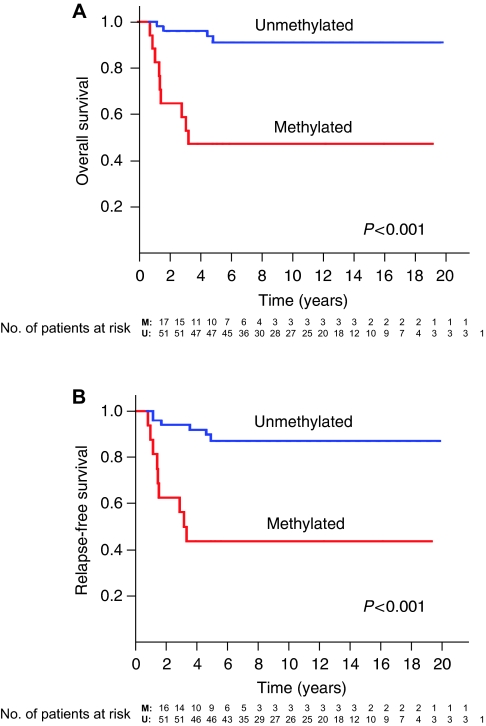
Kaplan–Meier survival curves of 68 neuroblastoma patients: correlation of pretherapeutic serum *RASSF1A* methylation status with overall survival (**A**) and relapse-free survival (**B**). M, methylated: patients with serum methylation of *RASSF1A*. U, unmethylated: patients without serum methylation of *RASSF1A.* The 5-year overall survival was more than 90% in patients without methylation, whereas lower than 50% in patients with methylation (*P*<0.001).

The association of pretherapeutic serum methylation status of *RASSF1A* with clinical outcome was analysed in 68 patients with known follow-ups. Univariate analyses revealed prognostic significance for age at diagnosis ⩾12 months, stage 4 and *MYCN* amplification (*P*=0.002, *P*<0.001 and *P*<0.001, respectively; Table 3) in this cohort, as expected. Patients with serum-methylated *RASSF1A* had significantly worse overall survival than patients with serum-unmethylated *RASSF1A* (*P*<0.001, log-rank test; Figure 4A). The 5-year survival was more than 90% in patients without serum methylation of *RASSF1A*, whereas lower than 50% in patients with serum methylation of *RASSF1A* (hazard ratio, 9.2; 95% confidence interval (95% CI), 2.8–30.1; *P*<0.001); Table 3). *RASSF1A* methylation in serum and the known prognostic factors were also correlated with relapse-free survival as well as with overall survival (*P*<0.001; Table 4; Figure 4B). Furthermore, a subset analysis revealed that stage 3 patients also had a trend towards poorer prognosis when *RASSF1A* was methylated in serum. When limited to cases in stage 3, two of the three patients with serum-methylated *RASSF1A* died, whereas all eight patients with serum-unmethylated *RASSF1A* are alive (*P*=0.055, Fisher's exact test). In a multivariate analysis including age, sex and tumour stage, serum *RASSF1A* methylation was still associated with poor outcome with a hazard ratio of 2.4 (95% CI, 0.6–9.2), although this did not reach statistical significance (*P*=0.194; Table 5).

## Discussion

In patients with malignancies, aberrant methylation of serum DNA has been reported (Müller *et al*, 2003; Ibanez de Caceres *et al*, 2004; Fiegl *et al*, 2005; Mori *et al*, 2005; Koyanagi *et al*, 2006). We have detected cell-free tumour DNA in serum of neuroblastoma patients (Gotoh *et al*, 2005). Prognosis in stage 4 neuroblastoma patients with metastases is poor despite intensive chemotherapy (Maris *et al*, 2007). Therefore, this study aimed to explore the possible prognostic significance of aberrant promoter hypermethylation of *RASSF1A*, which has been found frequently in neuroblastoma tumours, using pretherapeutic serum of neuroblastoma patients as a surrogate marker for circulating tumour cells.

We first investigated the *RASSF1A* methylation status in 68 neuroblastoma tumour DNA samples in comparison with matched serum DNA samples. The methylation of *RASSF1A* was observed in this study in 94% of primary tumours. Our results show that promoter hypermethylation of *RASSF1A* occurs at a high frequency in primary neuroblastoma tumours and no correlation between *RASSF1A* methylation and known prognostic factors including stage, age and *MYCN* amplification, or outcome was seen. The high proportion of *RASSF1A* promoter methylation in tumours agrees with earlier reports in the literature, which have found *RASSF1A* to be hypermethylated in 52–94% of tumour DNA samples (Astuti *et al*, 2001; Harada *et al*, 2002; Wong *et al*, 2004; Yang *et al*, 2004, 2007; Banelli *et al*, 2005; Lazcoz *et al*, 2006; Michalowski *et al*, 2008). Several earlier studies with one exception (Yang *et al*, 2004) failed to find a statistical correlation between *RASSF1A* methylation in tumours and poor outcome (Astuti *et al*, 2001; Harada *et al*, 2002; Banelli *et al*, 2005; Michalowski *et al*, 2008). We also did not observe any relationship between *RASSF1A* methylation in tumours and prognosis. *RASSF1A* hypermethylation in tumours can be a relatively early event in neuroblastoma tumorigenesis as it is detectable in non-advanced early-stage tumours with high frequency. Although the prognostic significance of epigenetic changes of single genes in neuroblastoma tumour DNA remain controversial, a few studies have indicated that poor prognosis is associated with the CpG island methylator phenotype (Abe *et al*, 2005; Banelli *et al*, 2005; Yang *et al*, 2007), suggesting that aberrant methylation of multiple genes is likely to contribute to neuroblastoma pathogenesis.

As a next step, we analysed *RASSF1A* methylation status in 68 paired serum DNA samples. In contrast to tumours, *RASSF1A* methylation was detected in neuroblastoma patient serum from only 25% (17 out of 68). To investigate the clinical significance of the serum *RASSF1A* methylation, associations with established prognostic factors and outcome were evaluated. *RASSF1A* methylation in serum was found to be statistically associated with established prognostic factors. Serum *RASSF1A* methylation was more frequently detected in neuroblastoma patients with age ⩾12 months at diagnosis (*P*=0.002), stage 4 (*P*<0.001) and *MYCN* amplification (*P*<0.001). Furthermore, the presence of methylation of *RASSF1A* in serum was associated with poorer outcome. The influence of serum *RASSF1A* methylation on prognosis was found to be comparable with that of the currently most reliable marker, *MYCN* amplification in univariate analysis. A subset analysis of stage 3 patients showed a trend associating poor survival with serum *RASSF1A* methylation (*P*=0.055), although the data were limited due to the small number of patients in the subgroup. In multivariate analysis of survival, methylation of *RASSF1A* in serum had a hazard ratio of 2.42, but this association did not reach statistical significance (*P*=0.194). Further validation studies using a larger set of patients are necessary to confirm our findings.

The presence of tumour-derived DNA within the blood stream has been identified earlier (Müller *et al*, 2003; Fiegl *et al*, 2005; Mori *et al*, 2005). Recently, one study showed that the detection of circulating tumour cells was correlated with tumour-related methylated DNA in patients with melanoma (Koyanagi *et al*, 2006), suggesting that circulating tumour cells are a potential source of circulating methylated DNA. Our study suggests that methylated *RASSF1A* DNA in serum is a surrogate marker for circulating neuroblastoma cells. Another recently published study showed that *RASSF1A* methylation was also detectable in ovarian cancer patient’s serum at a high frequency from methylated tumour cases including several stage I tumours (Ibanez de Caceres *et al*, 2004). In the earlier study, there was no statistical association between tumour stage and positive detection in serum. However, some other studies have shown limited detection of *RASSF1A* methylation in the serum of patients with other neoplasms (Murray *et al*, 2004; Hesson *et al*, 2007). These differing results may suggest that free neoplastic DNA from ovarian cancer can access the blood stream more readily than that from other neoplasms including neuroblastoma.

In conclusion, this is the first study to examine epigenetic changes in a tumour suppressor gene, *RASSF1A*, the promoter of which is hypermethylated at a high frequency in neuroblastoma tumours, using serum DNA in a cohort of neuroblastoma patients. This study demonstrates the utility of detecting circulating methylated *RASSF1A*, which can be measured in serum, as a potentially predictive marker of neuroblastoma outcome. *RASSF1A* methylation in serum could have useful clinical applications in neuroblastoma management, if our results are confirmed in larger studies. However, we should not forget the limitation when attempting to translate our findings into the clinical fields as highly sensitive methylation analysis could be tricky because of incomplete bisulphite conversion by inexperienced analysts.

## Figures and Tables

**Table 1:** Characteristics of patients

**Characteristic**	**No. of patients (%)**
*Sex*	
Male	27 (39.7)
Female	41 (60.3)
	
*Age at diagnosis*	
<12 months	42 (61.8)
⩾12 months	26 (38.2)
	
*Stage*	
1	24 (35.3)
2	11 (16.2)
3	11 (16.2)
4	18 (26.5)
4S	4 (5.9)
	
*MYCN*	
Non-amplified	56 (82.4)
Amplified	12 (17.6)
	
*Diagnosis*	
GNB	7 (10.3)
NB	61 (89.7)
	
*Serum RASSF1A*	
Unmethylated	51 (75.0)
Methylated	17 (25.0)

**Table 2:** Associations between clinical factors and serum *RASSF1A* methylation status

**Characteristic**	**Methylated no.**	**Unmethylated no.**	**Total no.**	***P*-value**
*Age at diagnosis*				0.002
<12 months	5	37	42	
⩾12 months	12	14	26	
				
*Stage*				<0.001
1/2/4S	3	36	39	
3	3	8	11	
4	11	7	18	
				
*MYCN*				<0.001
Non-amplified	5	51	56	
Amplified	12	0	12	

**Table 3:** Univariate analysis of survival

**Characteristic**	**Hazard ratio**	**95% CI**	***P*-value**
*Age at diagnosis*			
<12 months	Reference		
⩾12 months	23.6	3.1–181.9	0.002
			
*Sex*			
Male	Reference		
Female	1.0	0.3–3.0	0.983
			
*Stage*			
1/2/3/4S	Reference		
4	19.8	4.4–89.5	<0.001
			
*MYCN*			
Non-amplified	Reference		
Amplified	8.2	2.7–24.7	<0.001
			
*Serum RASSF1A*			
Unmethylated	Reference		
Methylated	9.2	2.8–30.1	<0.001

CI=confidence interval.

**Table 4:** Univariate analysis of relapse

**Characteristic**	**Hazard ratio**	**95% CI**	***P*-value**
*Age at diagnosis*			
<12 months	Reference		
⩾12 months	12.5	2.8–55.3	<0.001
			
*Sex*			
Male	Reference		
Female	1.0	0.4–2.9	0.972
			
*Stage*			
1/2/3/4S	Reference		
4	14.3	4.0–51.0	<0.001
			
*MYCN*			
Non-amplified	Reference		
Amplified	7.2	2.6–20.0	<0.001
			
*Serum RASSF1A*			
Unmethylated	Reference		
Methylated	6.8	2.4–19.1	<0.001

CI=confidence interval.

**Table 5:** Multivariate analysis of survival

**Characteristic**	**Hazard ratio**	**95% CI**	***P*-value**
*Age at diagnosis*			
<12 months	Reference		
⩾12 months	1.2	1.0–1.5	0.066
			
*Sex*			
Male	Reference		
Female	0.6	0.1–2.5	0.452
			
*Stage*			
1/2/3/4S	Reference		
4	8.4	1.5–46.4	0.014
			
*Serum RASSF1A*			
Unmethylated	Reference		
Methylated	2.4	0.6–9.2	0.194

CI=confidence interval.
